# Efficacy, Safety, and Reliability of the Single Anterior Approach for Subaxial Cervical Spine Dislocation

**DOI:** 10.7759/cureus.34787

**Published:** 2023-02-08

**Authors:** Sharif Jonayed, Abdullah Al Mamun Choudhury, Md. Shah Alam, OZM Dastagir

**Affiliations:** 1 Orthopaedics and Traumatology, National Institute of Traumatology and Orthopaedic Rehabilitation (NITOR), Dhaka, BGD; 2 Spine Surgery, National Institute of Traumatology and Orthopaedic Rehabilitation (NITOR), Dhaka, BGD; 3 Spine Surgery, Bangladesh Spine and Orthopaedic General Hospital, Dhaka, BGD

**Keywords:** traumatic cervical spine injury, anterior cervical corpectomyand fusion (accf), subaxial cervical spine dislocation (csd), anterior cervical discectomy and fusion (acdf), single anterior approach

## Abstract

Background

Though there is ongoing controversy regarding the best treatment option for cervical spine dislocation (CSD), anterior cervical surgery with direct decompression is becoming widely accepted. However, managing all cases of subaxial CSD entirely by a single anterior approach is rarely seen in the published literature.

Methods

The study comprised patients with subaxial CSD who underwent surgical stabilization utilizing a single anterior approach. Most of the CSD was reduced and anterior cervical discectomy and fusion (ACDF) were performed. Anterior cervical corpectomy and fusion (ACCF) were done in unreduced dislocations. The patient's neurological condition, radiological findings, and functional outcomes were assessed. SPSS version 25.0 (IBM Corp., Armonk, NY) was used for statistical analysis.

Results

The total number of operated cases was 64, with an average of 42 months of follow-up. The mean age was 34.50±11.92 years. The most prevalent level of injury was C5/C6 (57.7%). Reduction was achieved in 92.2% of cases; only 7.8% of patients needed corpectomy. The typical operative time was 84.25±9.55 minutes, with an average blood loss of 112.12±25.27 ml. All cases except complete spinal cord injury (CSI) were improved neurologically (87.63%). The mean Neck Disability Index (NDI) was 11.14±11.43, and the pre-operative mean visual analog score (VAS) was finally improved to 2.05±0.98 (P<0.05). In all cases, fusion was achieved. The most common complication was transient dysphagia (23.4%). After surgery, no patient developed or aggravated a neurological impairment. Implant failure was not observed at the final follow-up except for two cases where screws were pulled out partially.

Conclusion

Based on the results of this study, a single anterior approach is a safe and effective procedure for subaxial CSD treatment with favorable radiological, neurological, and functional outcomes.

## Introduction

Injury to the cervical spine is a terrible event that can result in significant debility and even death if not treated properly. It occurs in 2-6% of all blunt trauma scenarios, of which 55% have related spinal cord damage and 10% to 25% may deteriorate later [1]. Road traffic accidents (RTAs) and falls are the most common causes. The risk factors for cervical injury are increasing age and male gender, and instant death may reach up to 20% in elderly patients [2].

Unless contraindicated, supervised axial traction is the cornerstone of initial treatment for reducing acute cervical spine dislocation (CSD) and realigning the segment. The currently recognized strategies for the treatment of CSD differ. Depending on the injury, instability, and cord compression, treatment options range from orthosis to surgical decompression and internal fixation [3]. The foremost intention of surgical intervention is to decompress the neural elements and stabilize the injured vertebra, thus reestablishing standard configuration. Surgery helps attain maximum utility of the compromised area, and other benefits include minor pain, neural improvement, and impending debility prevention. It also permits early retrieval and mobilization, helps treat other damages, promotes rehabilitation, and hastens patients' return to their daily lives [4].

Recent breakthroughs in cervical spine instrumentation and surgical procedures show substantial controversy regarding the best surgical method [5]. The literature recommends that it depends on the preferences and experiences of the surgeon. The main decisive factor is choosing closed reduction among conscious individuals or open reduction by anterior, posterior, or combined approach. Furthermore, it is widely recognized that the reduction method itself may aggravate the spinal cord injury, most commonly with a herniated or fractured disc dislodgment [6-8].

Even though both anterior and posterior techniques effectively treat cervical spine dislocation, the anterior approach has become more widespread over the past 10 years [9-13]. The anterior approach is relatively atraumatic with low morbidity and lower complication rate and can be done in a supine position. Additionally, the anterior approach eliminates disc fragments displaced into the spinal canal. It allows treating only one motion section, resulting in fewer neck problems [14,15], whereas, for achieving the same realignment in the posterior approach, more than one motion segment needs to be fused; this is associated with the hazards of undue dissection, postsurgical pain, blood loss, and problems preserving safe vital signs in the prone position [5,16].

Meanwhile, kyphosis of the afflicted segment occurs during standalone short-level posterior fixation due to subsequent disc degeneration with observed increased instability. Again, fixation through combined approaches is quite troublesome. The main disadvantages are prolonged operation time, increased bleeding, poor wound healing, and difficulty in adjusting posture.

Considering all these factors, we have opted to manage all cases of CSD in a simplified manner by a single anterior approach; in this paper, we will evaluate the efficacy, safety, and reliability of this technique.

## Materials and methods

After receiving IRB approval, the study was conducted at the National Institute of Traumatology and Orthopaedic Rehabilitation (NITOR). The prospective case series included 64 patients (16-73 years) with cervical spine dislocation (either unilateral or bilateral) within three weeks of injury who underwent anterior cervical discectomy and fusion (ACDF) or anterior cervical corpectomy and fusion (ACCF) using a tricortical bone graft from the iliac crest and were stabilized by cervical plate and screws from July 2014 to June 2019. The study excluded patients with pathological fractures, polytrauma, extreme age (<16 years >70 years), and head injury.

The patients signed informed written consent forms that detailed the operation and treatment options. The patients were initially resuscitated according to the advanced trauma life support (ATLS) protocol in the emergency department. After initial stabilization with a rigid cervical collar, a comprehensive history and examination was recorded (sex, age, mechanism of injury, comorbidities, and occupation). The American Spinal Injury Association (ASIA) Impairment Scale was used to classify pre-operative neurological conditions. In every case, plain X-rays of the cervical spine were taken. An immediate supervised awake traction was performed without delay, and neurology was constantly watched for any worsening. Initially, a 10kg to 15kg weight was employed, gradually increasing by 2.5 kg every six to eight hours until reduction was achieved. In traction, serial X-rays were taken. Later, a computed tomography (CT) scan, magnetic resonance imaging (MRI), or 3D CT scan was obtained in selected cases. The patient received surgery on the following available schedule. 

**Figure 1:**
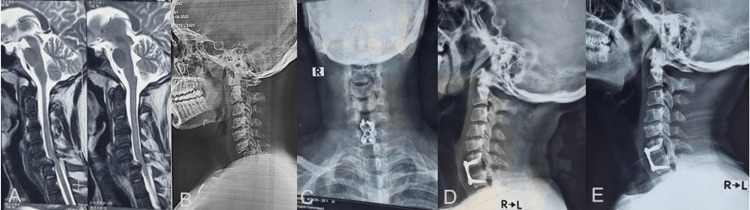
Anterior cervical discectomy and fusion (ACDF) A. Preoperative T2-weighted sagittal section of the cervical spine B. Preoperative X-ray of the cervical spine in lateral view C: Postoperative X-ray of the cervical spine in the antero-posterior and (D) lateral view E: X-ray of the cervical spine in lateral view at final follow-up (fusion)

Figure 1 shows a preoperative X-ray and MRI along with immediate postoperative and final X-rays of ACDF.

**Figure 2:**
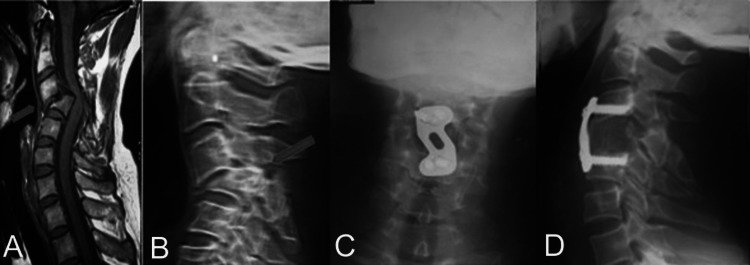
Anterior cervical corpectomy and fusion (ACCF) A: T1-weighted sagittal MR image of the cervical spine showing fracture-dislocation at C3/4 B: X-ray cervical spine in lateral view showing fracture-dislocation at C3/4 C: X-ray cervical spine in antero-posterior and (D) lateral view showing solid fusion mass at final follow-up

Figure 2 shows the preoperative X-ray and MRI along with immediate postoperative and final X-rays of ACCF.

Data such as operation time, blood loss, and intraoperative complications were recorded. Patients were mobilized as soon as their conditions permitted, usually the next day. All patients wore a stiff cervical collar for three weeks before switching to a soft collar for the next three weeks. For the first three months, patients were seen in the outpatient department (OPD) every two weeks, then monthly for the next three months, and lastly, every six months. Visual analogue score (VAS), Neck Disability Index (NDI) [17], Bridwell Fusion Index (BFI), and ASIA grading were done at each follow-up. To determine implant fixation and fusion, X-rays were taken. Successful fusion criteria include bridging trabecular bone, the absence of a radiolucent gap between the graft and endplate, and a gap of 2 mm on flexion and extension views. SPSS 25.0 (IBM Corp., Armonk, NY) was used to evaluate the data collected on a proforma.

The same surgeon and anesthesiologist performed all procedures. A trained technician and an experienced data analyst gathered the required information and final statistical analysis. They were uninformed of the study's aims and the technique by which the patient was operated on.

We routinely used an anterior approach with a left-sided transverse incision for surgery. Patients who showed a dislocation reduction preoperatively (supervised awake traction) underwent ACDF. Those who showed no reduction in traction operative reduction were tried within the earliest possible time (24-48 hours) in the standard anterior approach. Casper screws were employed, and a discectomy was performed to achieve local kyphosis per-operatively. Then we removed the caudally placed Casper screw. In the empty disc space, a small periosteal elevator was inserted into the lower vertebra's posterior border, leveraging the superior vertebra. Then gentle manipulation was done by applying backward and forward pressure to the caudal vertebrae by the periosteal elevator while the cephalad vertebral body was pushed back. The procedure was done by another assistant using the proximal Casper pin. Gentle traction and counter-traction was maintained during this whole procedure, while any baseline change in the somatosensory evoked potential (SSEP) and motor evoked potential (MEP) were recorded. After the reduction, ACDF was performed. Patients who showed no reduction at all by open maneuvers or reduced with recurrent instability underwent Anterior cervical corpectomy and fusion (ACCF)

**Figure 3:**
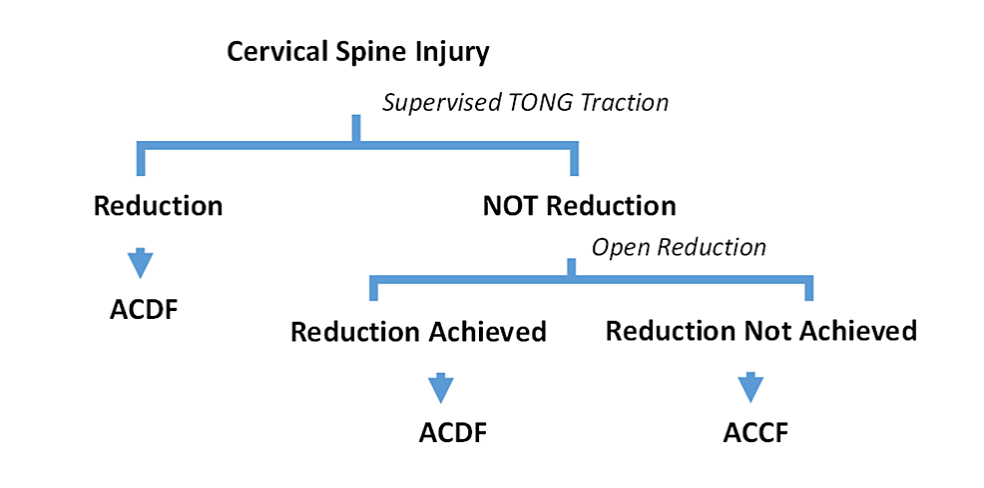
Schematic diagram of our preferred protocol ACDF: Anterior cervical discectomy and fusion ACCF: Anterior cervical corpectomy and fusion

Figure 3 shows a schematic diagram of our preferred method.

## Results

All cases were followed for 34 to 50 months (mean 42±8 months). The average age was 34.50±11.92 years (16-70 years). This study included 64 cases (M=58, F=6) in which the most common mechanism of injury was a fall while carrying a heavy weight on the head (51.6%), followed by falls from height (29.7%) and RTA (18.7%).

The study group consisted of 42 (65.6%) patients with bifacet dislocation and 22 (34.4%) with unifacet dislocation. The most typical level of involvement was C5/C6 (57.7%); after that C6/C7 (26.6%), C4/C5 (9.4%), and C3/C4 (6.3%).

Of 64 patients preoperatively, eight (12.5%) patients had ASIA A, 18 (28.1%) had ASIA B, 26 (40.6%) had ASIA C, and 12 (18.8%) had ASIA D neurology. Postoperatively, eight (12.5%) patients had ASIA A, four (6.3%) had ASIA C, 24 (37.5%) had ASIA D, and 28 (43.7%) had ASIA E neurology. A two-grade improvement was noted in 30 (46.9%) patients and a one-grade improvement in 26 (40.6%) patients. No improvement was noted in all patients with ASIA A (eight; 12.5%). One patient showed initial neurological deterioration from ASIA D to ASIA C, which may be due to manipulation that was improved to ASIA E at the last follow-up.

**Figure 4:**
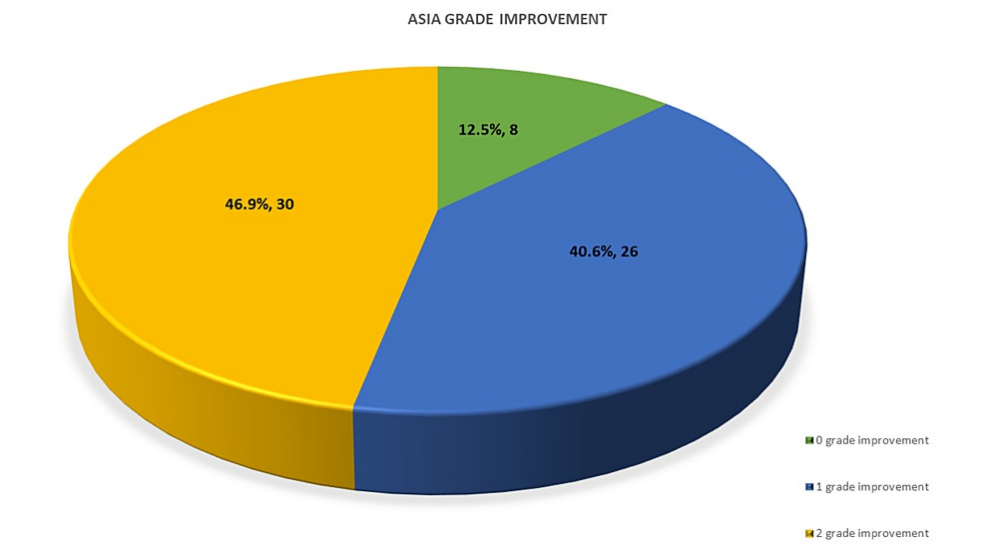
Neurological grade improvement ASIA: American Spinal Injury Association

Figure 4 shows neurological improvement after surgery.

Since the injury to operation, the average time was 7.9±4.2 days ranging from two to 19 days. The mean operative time was 84.25±9.55 minutes. Peri-operative blood loss was 112.12±25.27 ml. With regards to the reduction of maneuver, indirect reduction was performed in 33 (51.6%) patients, open reduction in 26 (40.6%), and corpectomy in five (7.8%).

The functional outcome was assessed using the VAS and the NDI. Mean pre-operative VAS (7.0±1.1) was improved to (2.05±0.98) at the final follow-up (p-value <0.05). According to the NDI, 35 (54.6%) patients had a mild disability, 17 (26.6%) patients had no disability, four (6.3%) patients had a severe disability, and eight (12.5%) patients had a complete disability. The average NDI was 11.14±11.43.

A Pearson correlation coefficient determined the association between final VAS and NDI. There was a reasonably positive association between the two variables, r = 0.469, N=64, p=0.000, i.e. if the patient with less pain showed minimal disability.

Table 1 shows a correlation between the final VAS and NDI.

**Table 1:** Correlation between Final VAS and NDI **. Correlation is significant at the 0.01 level (2-tailed). VAS: Visual Analog Score NDI: Neck Disability Index

Correlations			
		Final Pain status	Functional Status
Final Pain status	Pearson Correlation	1	0.469^**^
	Sig. (2-tailed)		0.000
	N	64	64
Functional Status	Pearson Correlation	0.469^**^	1
	Sig. (2-tailed)	0.000	
	N	64	64

The Bridwell criteria were used to evaluate fusion. Of the 64 patients, 37 (57.8%) showed Grade I fusion, 23 (35.9%) showed Grade II fusion, and four (6.3%) showed Grade III fusion. The Pearson correlation coefficient was measured to detect the relationship between fusion status and NDI. There was a strong positive correlation between the two variables, r = 0.546, N=64, p=0.000. Table 2 depicts the correlation between fusion status and NDI.

**Table 2:** Correlation between fusion status and NDI **. Correlation is significant at the 0.01 level (2-tailed). NDI: Neck Disability Index

Correlations		Fusion status	Functional Status
Fusion status	Pearson Correlation	1	0.546^**^
	Sig. (2-tailed)		0.000
	N	64	64
Functional Status	Pearson Correlation	0.546^**^	1
	Sig. (2-tailed)	0.000	
	N	64	64

Table 3 shows complications following the procedures. The most common complication observed in our series is dysphagia in 15 patients, donor site pain in five cases, a dural tear in three cases, and superficial infection in two cases. We observed screws pulled out in two instances currently under sequential follow-up.

**Table 3:** Complications

Complications	Quantity	Valid percent
Dysphagia	15(55.6%)	23.4
Donor site pain	5(18.5%)	
Dural tear	3(11.1%)	
Superficial infection	2(7.4%)	
Screw pulled out	2(7.4%)	
No complications	49	76.6

## Discussion

Proper and timely treatment of subaxial CSD is of paramount importance. However, the decision on reduction and surgical approach is still being debated. Each strategy has its merits and limitations. The spine surgeon's goal is to select the best therapeutic strategy for each patient to achieve satisfactory results. In this study, we chose the anterior-only approach to treat all cases of CSD.

Most of the patients in our series were young adults with male predominance as compared to Laus et al. (1993) [18] & Singhal et al. (2008) [19].

Bearing load on the head was the most common cause (51.6%), followed by falls from height (29.7%). Abdelgawaad et al. (2020) [20] and Laus et al. [18] showed that the common cause of injury was RTA (55%) and (85%), respectively. The difference between these studies conducted in two different geographical regions was again due to the patient's socio-economic status. It indicates that people require more awareness about carrying loads on their heads and climbing trees to reduce the chances of injury.

The most involved level of the spine was C5/C6 (57.7%), followed by C6-C7 (26.6%). Similar results were observed by Singhal et al. [19], which were 53.7% and 28.1%, respectively.

For reduction, we had performed awake traction prior to MRI because obtaining an MRI could cause the reduction to be delayed. An MRI may take an entire day in our area of the world. So, we do not postpone reduction for MRI though cervical fracture-dislocation is frequently associated with disc disruption [21-23]. However, paralysis secondary to retropulsion fragments is rare. Although up to 22% of patients have been found to have significant disc herniation post-reduction, these do not correlate with neurologic deterioration [22]. None of our patients showed neurologic damage during awake reduction.

As per the literature review, indirect reduction could be achieved in most cases and was hence easily managed by the anterior approach only. A combined approach managed the unreduced dislocation. But, we did all our cases using a single anterior approach irrespective of reduction to formulate an algorithm in efficacy, safety, and reliability based on available resources and the paying capability of the patients. The main difference of this study from others is that we did not do posterior surgery for unreduced CSD because of late presentation or its association with pedicle fractures. Instead, a corpectomy was done to decompress the cord and maintain the normal cervical alignment. So, our procedure reduces surgical time and morbidity associated with the combined approach, avoids undue dissection, and minimizes cost. Around 92.2% of patients in our series experienced indirect or open reduction, comparable to Reindl et al. (95.12%) [24]. Failure of reduction was most commonly seen in unifacet dislocation.

In this investigation, the average operational time was 84.25±9.55 minutes, with a mean blood loss of 112.12±25.27 ml. The anterior method takes less time to perform than the posterior technique, with Kwon et al. (2007) [25] reporting a mean operative time of 103 minutes. Yukawa et al. (2009) [26] observed similar findings on the posterior route, with a mean operational time of 101 minutes and a mean blood loss of 190 ml. Obviously, the combined approach would take more time as well as would result in higher blood loss. This demonstrates that the anterior approach, which takes less time in the operating room, is appropriate for patients who cannot tolerate a long period of anesthesia or those with neurological impairments, as time is of the essence.

In terms of neurological outcome, there was no significant difference between anterior and posterior techniques compared to the literature data [25,23,27]. Brodke et al. (2003) [28] found no significant difference between methods, with 70% and 57% of individuals with poor neurology improving when treated anteriorly and posteriorly, respectively. This is why the anterior-only approach to manage all cases of subaxial CSD in a simplified manner was chosen.

Most of the patients presented in our series were ASIA C (26; 40.6%) patients, followed by ASIA B in 18 (28.1%) patients, signifying the magnitude of neurological deficit. From a neurological point of view, two-grade improvement was noted in 30 (46.9%) patients, followed by one-grade improvement in 26 (40. 63%) patients, and all patients with ASIA A showed no neurological recovery. Mcafee et al. (1995) [29] observed a shift of ASIA grade 1 in 76% of cases and a change of ASIA grade 2 in 12% of cases. We observed only one case had a transient neuro-deficit that was recovered fully and improved neurologically at the final follow-up, as seen by Mcafee et al [29]. It indicates the safety of the single anterior approach.

In terms of functional outcome, the mean VAS improved to 2.05±0.98 at the last follow-up, which was statistically significant at paired sample correlations. In 52 (81.3%) instances, NDI scoring revealed minimal to no disability. The remaining cases had severe (four cases of ASIA C) to complete (eight instances of ASIA A) disability. But that did not have an impact on mean NDI. Furthermore, it was discovered that the status of anatomical reduction and accurate imaging measures did not significantly correspond with self-reported outcome measures.

At the final follow-up, fusion was achieved in all cases irrespective of neurology or procedures (ACDF/ACCF). Its functional effects are directly related to the fusion rate. The Neck Disability Index score was highly linked to neurological and radiological outcomes.

Implant failure, graft dislodgement, hematoma, revision, and deformity are uncommon complications [23]. We did not encounter any significant issues. Implant failure was not observed at the final follow-up except for two cases where the patients' screws were partially pulled out and are currently under constant supervision. At two weeks, the most common problem we found was mild dysphagia in 15 (23.4%) patients. In the first two weeks, the reported incidence was around 71% [22]. Next was donor site pain in five (7.8%) patients and dural injury in three (4.7%) managed accordingly with no CSF fistula or pseudomeningocele formation. Aronson et al. (1968) [30] also reported temporary dysphagia (4.7%) and temporary hoarseness (2.32%) in 100 patients; dura was injured in one (8.3%) patient, which is 11% in Brodke et al [28].

Limitations of the study are that this is a case series with a smaller sample size and a single-center design.

## Conclusions

Most subaxial cervical facet dislocation can easily be reduced and treated by ACDF. Corpectomy can also effectively manage unreduced dislocation to avoid an extensive combined approach. So, a single anterior approach is a good alternative compared with other combined procedures, i.e., anterior-posterior, posterior-anterior, or posterior-anterior-posterior. Combined approaches require implants placed both anteriorly and posteriorly, therefore the cost is higher. In our preferred method, implants were used only anteriorly. It is technically more accessible, less traumatic, relatively safe, and an effective procedure with an excellent neurological and radiological outcome with a low complication rate. It significantly shortens the operation time as well as the rehabilitation period. Considering all these factors, a single anterior approach could effectively manage all cases of subaxial CSD.
